# Scorpion toxins targeting Kv1.3 channels: insights into immunosuppression

**DOI:** 10.1590/1678-9199-JVATITD-1481-18

**Published:** 2019-04-15

**Authors:** Isadora S Oliveira, Isabela G Ferreira, Gabriel M Alexandre-Silva, Felipe A Cerni, Caroline M Cremonez, Eliane C Arantes, Umberto Zottich, Manuela B Pucca

**Affiliations:** 1 School of Pharmaceutical Sciences of Ribeirão Preto, Department of Physics and Chemistry, University of São Paulo, Ribeirão Preto, SP, Brazil.; 2 Medical School, Federal University of Roraima, Boa Vista, RR, Brazil.; 3 Ribeirão Preto Medical School, Department of Biochemistry and Immunology, University of São Paulo, Ribeirão Preto, SP, Brazil.

**Keywords:** voltage-gated potassium channels, Kv1.3, scorpion toxins, KTx, immunosuppression

## Abstract

Scorpion venoms are natural sources of molecules that have, in addition to their toxic function, potential therapeutic applications. In this source the neurotoxins can be found especially those that act on potassium channels. Potassium channels are responsible for maintaining the membrane potential in the excitable cells, especially the voltage-dependent potassium channels (Kv), including Kv1.3 channels. These channels (Kv1.3) are expressed by various types of tissues and cells, being part of several physiological processes. However, the major studies of Kv1.3 are performed on T cells due its importance on autoimmune diseases. Scorpion toxins capable of acting on potassium channels (KTx), mainly on Kv1.3 channels, have gained a prominent role for their possible ability to control inflammatory autoimmune diseases. Some of these toxins have already left bench trials and are being evaluated in clinical trials, presenting great therapeutic potential. Thus, scorpion toxins are important natural molecules that should not be overlooked in the treatment of autoimmune and other diseases.

## Background

Venomous animals are specialized predators that developed high degree of complexity compounds for their own biological purposes [1]. They produced venoms that contain a cocktail of toxins with a great structural and functional diversity [2]. In addition, venom components present diverse characteristics including low-molecular mass, stability, high potency and selectivity for a wide variety of targets in mammalian systems [3, 4].

Nevertheless, one important advantage of venoms is that they can act on many target molecules used for therapeutic intervention [5, 6]. Therefore, animal venoms are increasingly recognized as a new emerging source of peptide-based therapeutics [2, 7, 8].

Scorpion venoms are composed of numerous toxins, mostly of peptides and neurotoxins, which can interfere with all biological systems [9, 10]. Moreover, neurotoxins are known to modify cell membrane ion channels and to cause the release of massive neurotransmitters and cytokines [10, 11]. In this sense, scorpion venoms have been considered as invaluable resources of ion channel inhibitors and/or modulators, and potassium channel acting toxins (KTx) are one of the most studied [9, 12-14].

Voltage-gated potassium channels (Kv) have received much attention because they are widespread in almost all tissues, besides presenting high expression in mammalian cells. They are involved in the regulation of many physiological processes, including heart rate, neuronal excitability, insulin production, epithelial electrolyte transport, cell proliferation, smooth muscle contraction, neurotransmitter release and immune response [15]. In particular, the voltage-gated potassium channel type 1.3 (Kv1.3) is a well-recognized functional marker and an attractive pharmacological target for treating autoimmune diseases [16]. Thus, based on the interactions between scorpion toxins and potassium channels, a great effort has been employed to find scorpion toxins selectively acting on the Kv1.3 channels [17]. It is important to emphasize that other animals also produce toxins with action on Kv1.3, such as ShK toxin, a potent Kv1.3 inhibitor obtained from *Stichodactyla helianthus* Caribbean sea anemone venom, and also named as Dalazatide (its synthetic analog) [18]. This first-in-class investigational drug completed phases Ia and Ib of clinical trials and phase IIa is expected to start in this year (2018) [19]. Clinical studies demonstrated that Dalazatide (Kineta Inc.) can decrease skin lesions of psoriasis [20]. Also, this molecule is being study for bringing novel new therapeutics of other autoimmune diseases (e.g. multiple sclerosis and rheumatoid arthritis) [21, 22].

This review will present the current state of art regarding scorpion toxins targeting Kv1.3 channels. Moreover, it will provide details on the Kv1.3 discovery, structure and function focusing on its important role as a target to induce immunosuppression.

## Kv1.3 channel discovery and structure

Kv1.3 currents were firstly recorded in both resting and mitogen stimulated human T lymphocytes in 1984, using patch-clamp technique [23], although in that time the channel did not received the Kv1.3 nomenclature. In the following year, its biophysical properties were also reported. Kv1.3 demonstrated to be a delayed rectifier like voltage-gated potassium channel presenting a slow and very complex mode of inactivation: the lymphocyte K^+^ currents turn on with a sigmoid time course upon the depolarization and, then inactivate almost completely with voltage dependence and kinetics that resemble delayed rectifier K^+^ channel of muscle and nerve [24].

The name Kv1.3 was only introduced in 1991 with a standardized nomenclature [25]. In that study, researchers demonstrated through phylogenetic trees that the channel presents a significant structural homology with the *Shaker* channel from *Drosophila melanogaster*. Therefore, the Kv1.3 channel was classified as a mammalian *Shaker*-related voltage-gated potassium channel encoded by the KCNA3 gene, which is found in humans, rats and mice [25, 26].

The functional channel is formed by four pore loop-containing α-subunits arranged as a homotetrameric association. Each monomer contains 528 amino acids, six transmembrane segments (S1 through S6) and one pore region formed by the interactions of the four homotetramer to create the walls. In this pore, both the N-terminal and C-terminal domains are located within the cytoplasm [27].

The N-linked glycosylation site is in the external S1-S2 loop, the channel presents two protein kinase C sites that are located in the S4-S5 linker and a tyrosine kinase site in the N-terminal region. The tripeptide sequence motif G(Y/F)G located in the S5-S6 linker is common to the pore or P loop of these channels and, four of the pore loop domains contribute to the formation of a functional K^+^ conducting pore [27-29].

The transmembrane S4 segment represents the major component of the voltage sensor for this channel, it contains positively charged residues (like lysine or arginine) at almost every third portion of its structure [29].

One interesting approach employed for studying the structure of the Kv1.3 channels is the use of peptides such as scorpion toxins, for mapping the external vestibule of the channel. Based on that, a study was conducted using four scorpion toxins that have the capacity to block the channel (kaliotoxin, charybdotoxin, margatoxin and noxiustoxin). The authors used mutagenesis in combination with quantitative analytical methods and, they could identify several channel residues that interact specifically with toxin residues. These studies revealed the existence of a shallow (4-6 Å) and wide (35 Å) saucer-shaped external vestibule with the K^+^ channel signature sequence (GYGD) forming a through as its center [30, 31].

One unique feature of the Kv1.3 external vestibule is the presence of a ring of histidine ~9-14 Å in diameter, positioned at the outer entrance of the ion conduction pathway and, none of the so far known Kv channels has a histidine at this position [28].

The external entry to the channel pore consisting of portions of the P-loop and adjacent residues in S5 and S6 segments constitutes possible binding sites for toxins and K^+^ channel blockers. Studies demonstrated that toxins are observed to lock into the channel selectivity filter through a lysine residue in the position 27, performing a strong interaction with the channel selectivity filter. Although most of the toxins bind to the Lys-27, this is not a rule, some interact differently with the channel peripheral acidic residues such as Glu-420, Asp-423 and Asp-433, or can even present a different Lys position (Lys-25) [32-34].

## Kv1.3 channel role

Kv1.3 channels are molecules expressed in macrophages, microglia cells, natural killer cells, B and T lymphocytes, osteoclasts, platelets, central nervous system (CNS), prominent in the olfactory bulb and testis [35-38] being able to participate in numerous physiological processes [39]. These channels are important for maintaining the negative membrane potential as they promote intracellular K^+^ efflux as well as help in the influx of extracellular Ca^2+^ through Ca^2+^ channels activated by the release of Ca^2+^ [40-44]. However, this rectifier channel has a characteristic that differentiates it from the other channels from Kv1 family, which is its inactivation for long periods (~ 1 minute or more) after repeated depolarization [45, 46]. Blockage of these channels may bring improvements related to homeostasis regulation, as well as to the immune system [43, 47]. Therefore, mice that have the absence of these channels (knockout animals or Kv1.3^-^/^-^) present variation on signaling molecules levels, such as postsynaptic density protein 95 (PSD-95) and tropomyosin receptor kinase B (TrKB) [48], as well as, there is a decrease of some markers, such as somatostatin and neuropeptide Y interneurons, and an increase of parvalbumin on the cerebral cortex [45]. Moreover, these murine model presents neuroprotection against experimental autoimmune encephalomyelitis (EAE) and increases the levels of interleukin-10 (IL-10) production [49], besides the expression of high levels of forkhead box protein O1 (FoxO1), phosphospecific signal transducer and activator of transcription 5 (pSTAT5), cytotoxic T-lymphocyte-associated protein 4 (CTLA4), erythroid transcription factor (GATA1) and interleukin-2 receptor alpha chain (CD25) [50]. On the other hand, Kv1.3^-/-^ rats show that maximal T-cell responses against autoantigen or repeated tetanus toxoid stimulations require both Kv1.3 and KCa3.1, since knockdown of Kv1.3 rats developed adjuvant-induced arthritis (AIA) in a manner similar to WT rats, indicating that there were no defects in T-cell activation [51].

In addition, it has already been shown that Kv1.3^-^/^-^ mice are able to discriminate odors, as well as, they present high olfactory function, being thus named "super-smellers" [48]. It was also found that these mice were resistant to obesity when induced by a moderately high-fat diet [52, 53]. They had irregular intake, as well as their metabolic activities, increased locomotion during the dark cycle and behaviors similar to hyperactivity [48, 54]. Recent studies have shown that the same mice model present exacerbated behaviors related to anxiety, in addition to being inattentive [55].

Although this phenotype causes all these differences, it has been observed that Kv1.3^-^/^-^ animals do not present anomalies in lymphocytes, both in number and type, in T cell proliferation and in thymocyte apoptosis, which may be related to the compensatory increase of the chloride current, maintaining the negative membrane potential [56].

In addition to these animal models, there are several pathological conditions where the dysregulation of Kv1.3 occurs [39, 57, 58]. The list below presents an onset of diseases in which Kv1.3 participates in their pathology (i.e. they are important for cells involved):

- Allergic contact dermatitis: skin disease T-cell mediated, as a delayed hypersensitivity reaction (type IV). In this pathology there is an increase of T_EM_ cells T-effector memory lymphocytes (T_EM_) [39, 59];
- Alopecia areata: an autoimmune disease that acts against the hair follicle, which is surrounded by T and natural killer (NK) cells, and T_EM_ cells [39, 60-62];
- Asthma: pathology characterized as chronic inflammation of the airways, caused by infectious or environmental stimuli, leading to reversible bronchoconstriction and presenting an increase of leukocytes [39, 63];
- Atherosclerosis: chronic inflammatory disease in which the innate and adaptive immune responses are activated, forming fatty streaks and the Kv1.3 channels being necessary for the foam cells [39, 64];
- Breast cancer: one of the most common types of cancer among females, but there are still controversies regarding Kv1.3 channels, which may be increased or decreased in tumors [39, 65];
- Chronic lymphocytic leukemia: type of chronic leukemia which there is an exacerbated production of B cells, with Kv1.3 channels being very expressed in cell membranes and mitochondria and, when inhibited, leads to cellular apoptosis [39, 66, 67];
- Chronic renal failure: loss of renal function, which occurs with chronic inflammation, with the proliferation of leukocytes in the kidneys that express large amounts of Kv1.3 channels [39, 68];
- Cognitive disabilities: this terminology is given to people who have some deficit of attention, perception, memory, conceptualization, perception, learning disabilities, autism, dyslexia among others related problems [69]. With the inhibition of Kv1.3 channels, an improvement in the symptoms of this disabilities is observed [39];
- Crohn’s disease: chronic inflammation of the gastrointestinal system, in which there is an increase of T_EM_ cells [39, 70];
- Multiple sclerosis: disease mediated by immune system, causing damage to the CNS, such as loss of axons and demyelination [41, 71]. In this pathology there is an increase of T_EM_ cells [18, 39];
- Muscle sarcomas: type of muscular system cancer, which Kv1.3 channels are expressed in tumors and this expression may be related to its severity [39, 72];
- Obesity: disease associated with human behavior, which the individual presents binge eating and inhibition of Kv1.3 channels led to an increase in insulin sensitivity [39, 73].
- Prostate cancer: type of cancer that is closely related to male death and Kv1.3 channels are poorly expressed in these tumors, thus, there is an inverse correlation of this channel expression and the aggressiveness of the cancer [39, 74].
- Psoriasis: a skin disease of chronic and recurrent character, presenting a proliferative increase of the epidermis, as well as its inflammation [75]. In this pathology there is an increase of T_EM_ cells [39];
- Rheumatoid arthritis: chronic inflammatory autoimmune disease of the joints and may be accompanied by inflammation in other organs that are not articulated or other chronic symptoms, and, in this pathology, there is an increase of T_EM_ cells [39, 76];
- Systemic lupus erythematosus: autoimmune disease, which chronic inflammation is present, as well as innate and adaptive immune responses. In this pathology there are several symptoms and an increase of T_EM_ cells [39, 64];
- Type I diabetes mellitus: pathological and autoimmune conditions mediated by T_EM_ cells. In this pathology the destruction of pancreatic β-cells occurs [39, 77];
- Type II diabetes mellitus: type of diabetes resistant to insulin and causes hyperglycemia, and when there is inhibition of Kv1.3 channels, an increase in insulin sensitivity occurs [39, 78];
- Ulcerative colitis: chronic inflammation of the gastrointestinal system, being that in the colon and rectum. There is exacerbated activity of T lymphocytes (CD4^+^ and CD8^+^), which express Kv1.3 channels [39, 70];
- Vasculitis: chronic inflammation that occurs in the vessel wall, which can affect the lumen as well as cause necrosis and ischemia of the same [79]. In this kind of inflammation, T_EM_ cells are involved [39].

Therefore, based on the importance of Kv1.3 in different diseases, the channel has become an important target to novel drug design. 

## Kv1.3 channel and T cell regulation

**Figure 1.**
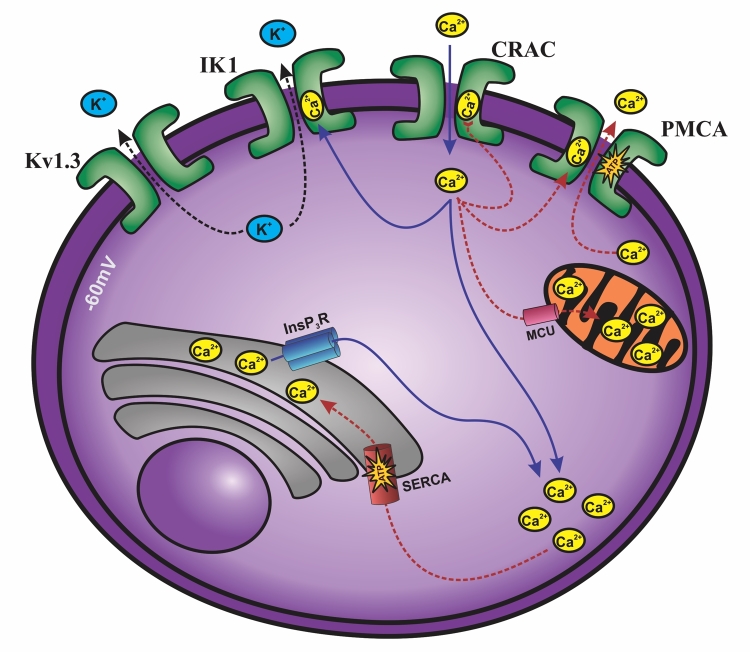
Kv1.3 and Ca^+2^ signaling in T cells. Depolarization of the T cells (about -60 mV) reduces the driving force for Ca^+2^ entry through calcium-release-activated calcium (CRAC) channels, which is counteracted by the opening of Kv1.3 channels. On the other hand, IK1 (intermediate conductance calcium-activated potassium channel protein 1) channels open in response to Ca^+2^ influx and increased intracellular Ca^+2^ concentration. Ca^+2^ are diminished by the ATP-dependent Ca^+2^ pumps - SERCA (sarco/endoplasmic reticulum Ca^+2^ ATPase) and PMCA (plasma membrane Ca^+2^ ATPase). PMCA is activated by increases in intracellular Ca^+2^ signals. Mitochondria can take up and store large amounts of Ca^+2^ from the cytoplasm using MCU (mitochondrial Ca^+2^ uniporter) or it can sequester Ca^+2^ locally. Iinositol-1,4,5-trisphosphate receptor (InsP3R) can also senses Ca^+2^ waves and enhance intracellular Ca^+2^ levels. Blue solid lines represent the pathways that enhance intracellular Ca^+2^ levels. Red dashed lines represent the pathways that reduce intracellular Ca^+2^ levels. Black dashed line represents K^+^ efflux.

Although many cells express Kv1.3, the most advanced studies with this channel are related to T cells. The mechanism that makes Kv1.3 channels important to T cells is directly related to intracellular calcium signaling (Fig. 1). Cellular depolarization increases calcium influx, a process that is counterbalanced by Kv1.3 channel opening, which repolarizes the cell and restores calcium through the calcium-release-activated-calcium channel (CRAC). Calcium signaling is also attenuated by the elimination of cytoplasmic cation by Ca^2+^ ATP-dependent endoplasmic reticulum (SERCA, sarco/endoplasmatic reticulum Ca^2+^ ATPase) and plasma membrane (PMCA, plasma membrane Ca^2+^ ATPase) pumps. PMCA pump is activated by increasing the concentration of intracellular Ca^2+^. Mitochondria play a double role in maintaining signaling via calcium: (*i*) it can sequester or store large amounts of Ca^2+^ from the cytoplasm through a single calcium channel (MCU, mitochondrial Ca^2+^ uniporter); (*ii*) MCU can still sequester the Ca^2+^ ions, inhibiting the negative regulation of CRAC channels [80]. All the calcium influx allows the nuclear factor of activated T cells (NFAT) to translocate to the nucleus and initiate transcription, leading to IL-2 cytokine secretion, a cytokine required for lymphocyte activation and proliferation [81, 82]. Thus, in the absence of sufficient Ca^2+^ influx via CRAC, T lymphocyte activation, proliferation, and effector functions are completely compromised, as demonstrated by a rare type of human immunodeficiency [83, 84].

**Figure 2.**
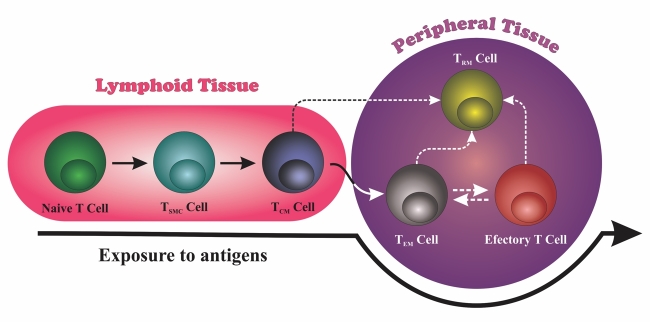
T cell subsets generation according to cellular activation and homing. T cells can be differentiated in different populations according to antigen exposure. Moreover, these T cells can be localized in different tissues (lymphoid and peripheral tissues). T_SCM:_ stem memory T cell. T_CM:_ central memory T cell. T_EM:_ effector memory T cell. T_RM:_ tissue resident memory T cell. Solid lines represent elucidated mechanism of differentiation. Dashed lines represent mechanisms still unclear.

All T cell types express Kv1.3, depending on their state of activation and differentiation [71]. According to cellular activation and homing, T cells can be classified into different phenotypes (Fig. 2): naive T cell, effector T cell and memory T cell. In addition, memory T cells may exhibit four different phenotypes: stem memory T cells (T_SCM_), central memory T cells (T_CM_), T_EM_ and tissue resident memory T cells (T_RM_) [85]. However, Kv1.3 channels have a greater expression and, consequently, a greater importance on effector memory T cells or T_EM_. Naïve cells and T_CM_ (CD4^+^ and CD8^+^) express 400 to 500 Kv1.3/cell. In contrast, T_EM_ cells express 1,500 Kv1.3/cell. In relation to the other potassium channel expressed in T cells, the potassium channel activated by calcium (KCa3.1), different numbers are observed: naive T and T_CM_: 200 to 500 KCa3.1/cell; T_EM_: 10 KCa3.1/cell) [41, 86, 87]. Thus, although both potassium channels (Kv1.3 and KCa3.1) are responsible for intracellular potassium signaling, differences in channel/cell numbers center the importance of the Kv1.3 channel for T_EM_ cells [88].

Knowing that T_EM_ cells are responsible for the development of autoimmune diseases, the studies with modulation of Kv1.3 channels have mightily intensified aiming new therapies using this receptor as target. Furthermore, because Kv1.3 channels are expressed in higher amounts only in T_EM_ cells, this targeted therapy would not impair the naïve or T_CM_ responses [41, 89, 90].

## Kv1.3 channel scorpion toxins’ blockers

Some scorpion venom toxins have the property of interacting specifically with K^+^ channels and are named as KTx [21, 91]. These toxins comprise a group of peptides having 23 to 64 amino acid residues, and may contain from three to four disulfide bonds [92]. According to their structure, amino acid sequence and disulfide bonds, these toxins can be classified, such as α, β, ε, κ, γ and λ-KTx [91-94].

Kumenskov and colleagues created a database that presents toxins that interact with K channels (kaliumdb.org). In that database there are 319 animal toxins, which, 174 are derived from scorpion venom. However, only 81 toxins are capable of interacting with the Kv1.3 channels (Table1) [95]. Although they present different affinities, we can define their potential to be considered a *bona fide* blocker by comparing them to the potency of Dalazatide, which presents an EC_50_ < 100 pM [96].

**Table 1.** Scorpion toxins targeting K_v_1.3 channels.

Toxin	Classification	Scorpion specie	Modification	Assay/Cell type	K_d_/IC_50_/EC_50_ (nM)	Reference
Aam-KTX	α-KTx 3.12	*Androctonus amoreuxi*	Kaliotoxin analog	Electrophysiological experiments with *Xenopus leaves* oocytes	1.1	[97]
ADWX-1*	α-KTx	*B. martensii*	Recombinant	Electrophysiological experiments with HEK293 cells	0.001	[98]
AgTX-1	α-KTx 3.4	*L. quinuqestriatus var. hebraeus*		Electrophysiological experiments with HEK293 cells	1.7	[99]
AgTX-2	α-KTx 3.2	*L. quinuqestriatus var. hebraeus*		Electrophysiological experiments with *Xenopus* oocytes/ *In vitro* L929 mouse fibroblast and Human T-lymphocytes	0.004	[99, 100]
Anuroctoxin*	α-KTx 6.12	*Anuroctonus phaiodactylus*		Electrophysiological experiments with human peripheral T lymphocytes	0.73	[101]
BmK86*	α-KTx 26.1	*Mesobuthus martensii* Karsch		Electrophysiological experiments with COS7 cells cells	150	[102]
BmKTT-1	δ-KTx 2.4	*Buthus martensii*	Recombinant	Electrophysiological experiments with HEK293 cells	129.7	[103]
BmKTT-2	δ-KTx 3.1	*Buthus martensii*	Recombinant	Electrophysiological experiments with HEK293 cells	371.3	[103]
BmKTT-3	δ-KTx 1.2	*Buthus martensii*	Recombinant	Electrophysiological experiments with HEK293 cells	> 1000	[103]
BmKTX*	α-KTx	*B. martensi Karsch*		Electrophysiological experiments with HEK293 cells	0.09	[104]
BmP01	α-KTx 8.2	*Mesobuthus martensii*		Electrophysiological experiments with *Xenopus leaves* oocytes	133.72	[105]
BmP02	α-KTx 9.1	*Mesobuthus martensii*		Electrophysiological experiments with *Xenopus* oocytes	7	[106]
BmP03	α-KTx 9.2	*Mesobuthus martensii*		Electrophysiological experiments with *Xenopus* oocytes	85.4	[106]
BmTX1	α-KTx 1.5	*Buthus martensii*		Electrophysiological experiments with *Xenopus* oocytes	1.5	[107]
BmTX2	α-KTx 1.6	*Buthus martensii*		Electrophysiological experiments with *Xenopus* oocytes	1.6	[107]
BoiTx1	α-KTx 3.10	*Buthus occitanus israelis*		Electrophysiological experiments with *Xenopus leaves* oocytes	3.5	[108]
BuTX	α-KTx 12.2	*Tityus trivittatus*		Electrophysiological experiments with *Xenopus leaves* oocytes	0.55	[34, 109, 110]
Ce1*	α-KTx 2.8	*Centruroides elegans*		Electrophysiological experiments with human T lymphocytes	0.7	[111]
Ce2*	α-KTx 2.9	*Centruroides elegans*		Electrophysiological experiments with human T lymphocytes	0.25	[111]
Ce3	α-KTx 2.10	*Centruroides elegans*		Electrophysiological experiments with human T lymphocytes	366	[111]
Ce4*	α-KTx 2.11	*Centruroides elegans*		Electrophysiological experiments with human T lymphocytes	0.98	[111]
Ce5	α-KTx 2.12	*Centruroides elegans*		Electrophysiological experiments with human T lymphocytes	69	[111]
Charybdotoxin	α-KTx 1.1	*L. quinquestriatus hebraeus*		Electrophysiological experiments with mammalian cell line that presents cloned Kv1.3 channels	2.6	[112]
CoTx1	α-KTx 10.1	*Centruroides noxius*		Electrophysiological experiments with Rat brain synaptosomes	5.3	[113]
Css20*	α-KTx 2.13	*Centruroides suffusus suffuses*		Electrophysiological experiments with human peripheral T lymphocytes	7.2	[114]
Ctri18*	α-KTx 15	*Chaerilus tricostatus*	Recombinant	Electrophysiological experiments with HEK293 cells	ND	[115]
Ctri9577*	α-KTx 15.10	*Chaerilus tricostatus*		Electrophysiological experiments with HEK293 cells	0.49	[116]
Ctry2908*	α-KTx 15	*Chaerilus tryznai*	Recombinant	Electrophysiological experiments with HEK293 cells	ND	[115]
Ctry68*	α-KTx 15	*Chaerilus tryznai*	Recombinant	Electrophysiological experiments with HEK293 cells	ND	[115]
Hemitoxin	α-KTx 6.15	*Hemiscorpius lepturus*		Electrophysiological experiments with *Xenopus* oocytes	2	[117]
Hetlaxin*	Data not shown	*Heterometrus laoticus*		Competitive binding experiments with chimeric KcsA-Kv1.3	410	[118]
HeTx204	κ-KTx 2.8	*Heterometrus petersii*		Electrophysiological experiments with HEK293 cells	ND	[119]
Hg1*	δ-KTx 1.1	*Hadrurus gertschi*	Recombinant	Electrophysiological experiments with HEK293 cells	6.2	[103, 120, 121]
Hongotoxin	α-KTx 2.5	*Centruroides limbatus*		Inhibition of ^86^Rb^+^ flux in HEK293 cells	0.086	[122]
HsTx1^#^	α-KTx 6.3	*Heterometrus spinnifer*		Electrophysiological experiments with L929 cells/ *In vivo* assay using DTH reaction, Lewis rat / *In vivo* assay using Pristane-induced arthritis model, Dark Agouti rats	0.011	[123, 124]
HsTx1[R14A]*	Data not shown	*Heterometrus spinnifer*	Synthetic peptide	Electrophysiological experiments with L929 cells	0.027	[124]
ImKtx1*	λ-KTx 1.1	*Isometrus maculates*		Electrophysiological experiments with HEK293 cells	1700	[125]
ImKTX88*^#^	α-KTx	*Isometrus maculates*	Recombinant	Electrophysiological experiments with HEK293 cells/ *In vivo* experimental autoimmune encephalomyelitis mice	0.091	[126, 127]
J123*	α-KTx 11.5	*Buthus martensii Karsch*		Electrophysiological experiments with HEK293 cells	0.79	[128]
Kaliotoxin^#^	α-KTx 3.1	*Androctonus mauretanicus mauretanicus*		Electrophysiological experiments with mammalian cell line that presents cloned Kv1.3 channels/ *In vivo* rat periodontal disease model	0.65	[112, 129]
Kbot1	α-KTx 9.5	*Buthus occitanus tunetanus*		Electrophysiological experiments with *Xenopus* oocytes	15	[130]
κ-Hefutoxin1	κ-KTx 1.1	*Heterometrus fulvipes*		Electrophysiological experiments with *Xenopus leaves* oocytes	40000	[131]
LmKTT-1a	δ-KTx 2.1	*Lychas mucronatus*	Recombinant	Electrophysiological experiments with HEK293 cells	> 1000	[103, 132]
LmKTT-1b	δ-KTx 2.2	*Lychas mucronatus*	Recombinant	Electrophysiological experiments with HEK293 cells	> 1000	[103, 132]
LmKTT-1c	δ-KTx 2.3	*Lychas mucronatus*	Recombinant	Electrophysiological experiments with HEK293 cells	> 1000	[103]
LmKTx10*	α-KTx 12.5	*Lychas mucronatus*		Electrophysiological experiments with HEK293 cells	28	[133]
LmKTx8*	α-KTx 11.4	*Lychas mucronatus*		Electrophysiological experiments with COS7 cells	26.4	[134]
Margatoxin*^#^	α-KTx 2.2	*Centruroides margaritatus*		Electrophysiological experiments with human peripheral T lymphocytes/ I*n vitro* human lung cancer A549/ *In vivo*-xenograft assay, nude mice	~0.030	[135, 136]
Maurotoxin	α-KTx 6.2	*Scorpio maurus palmatus*		Electrophysiological experiments with L929 cells	155	[123]
MegKTx3	α-KTx 16.7	*Mesobuthus gibbosus*		Electrophysiological experiments with *Xenopus leaves* oocytes	118.3	[137]
MeuKTx-1*	α-KTx 8.6	*Mesobuthus eupeus*		Electrophysiological experiments with *Xenopus leaves* oocytes	2.36	[105]
MeuKTx-3	α-KTx 3.13	*Mesobuthus eupeus*		Electrophysiological experiments with *Xenopus* oocytes	0.171	[138]
MeuKTx-4*	α-KTx 16.4	*Mesobuthus eupeus*		Electrophysiological experiments with *Xenopus leaves* oocytes	ND	[139]
MTX-HsTX1	α-KTx 6	*Scorpio maurus palmatus* and *Heterometrus spinnifer*	Chimera using maurotoxin and HsTX1 toxins	Electrophysiological experiments with L929 cells	4	[123]
Noxiustoxin	α-KTx 2.1	*Centruroides noxius*		Electrophysiological experiments with mammalian cell line that presents cloned Kv1.3 channels	1	[112]
OcyKTx2	α-KTx 6.17	*Opisthacanthus cayaporum*		Electrophysiological experiments with human peripheral T lymphocytes	17.7	[140]
OdK2*	α-KTx 3.1	*Odonthobuthus doriae*		Electrophysiological experiments with *Xenopus leaves* oocytes	7.2	[141]
OmTx1	κ-KTx 2.1	*Opisthacanthus madagascariensis*		Electrophysiological experiments with *Xenopus leaves* oocytes	ND	[142]
OmTx2	κ-KTx 2.2	*Opisthacanthus madagascariensis*		Electrophysiological experiments with *Xenopus leaves* oocytes	ND	[142]
OmTx3	κ-KTx 2.3	*Opisthacanthus madagascariensis*		Electrophysiological experiments with *Xenopus leaves* oocytes	ND	[142]
OsK1^#^	α-KTx 3.7	*Orthochirus scrobiculosus*		Electrophysiological experiments with L929 and murine erythroleukaemia cells/ *In vivo* neurotoxicity assay, C57/BL6 mice	0.014	[143]
PBTx1	α-KTx 11.1	*Parabuthus villosus*		Electrophysiological experiments with *Xenopus* oocytes	ND	[144]
PBTx3	α-KTx 1.10	*Parabuthus transvaalicus*		Electrophysiological experiments with *Xenopus leaves* oocytes	492	[144]
PEG-HsTX1[R14A]*	Data not shown	*Heterometrus spinnifer*	PEGylated molecule	Electrophysiological experiments with L929 cells	35.9	[124]
Pi1*	α-KTx 6.1	*Pandinus imperator*		Electrophysiological experiments with human lymphocytes	9.7	[145]
Pi2*	α-KTx 7.1	*Pandinus imperator*		Electrophysiological experiments with human lymphocytes	0.05	[145]
Pi3*	α-KTx 7.2	*Pandinus imperator*		Electrophysiological experiments with human lymphocytes	0.5	[145]
StKTx23*	α-KTx 30.1	*Scorpiops margerisonae*		Electrophysiological experiments with HEK293 cells	ND	[119]
Tc30	α-KTx 4.4	*Tityus cambridgei*		Electrophysiological experiments with T lymphocytes	16	[146]
Tc32	α-KTx 18.1	*Tityus cambridgei*		Electrophysiological experiments with T lymphocytes	10	[146]
Ts6^#^	α-KTx 12.1	*Tityus serrulatus*		Electrophysiological experiments with *Xenopus leaves* oocytes / *In vivo* assay using DTH reaction, BALB/c mice	0.55	[34]
Ts7	α-KTx 4.1	*Tityus serrulatus*		Electrophysiological experiments with *Xenopus leaves* oocytes	ND	[34]
Ts15^#^	α-KTx 21.1	*Tityus serrulatus*		Electrophysiological experiments with *Xenopus leaves* oocytes / *In vivo* assay using DTH reaction, BALB/c mice	508 1073	[147, 148]
Tst26*	α-KTx 4.6	*Tityus stigmurus*		Electrophysiological experiments with human peripheral T lymphocytes	10.7	[149]
Tt28	α-KTx 20.1	*Tityus trivittatus*		Electrophysiological experiments with *Xenopus leaves* oocytes	7.9	[150]
Urotoxin	α-KTx 6.21	*Urodacus yaschenkoi*		Electrophysiological experiments with human peripheral T lymphocytes	91	[151]
Vm23*	α-KTx 23.2	*Vaejovis mexicanus smithi*		Electrophysiological experiments with human peripheral T lymphocytes	10	[152]
Vm24*^#^	α-KTx 23.1	*Vaejovis mexicanus smithi*		Electrophysiological experiments with human peripheral T lymphocytes/ *In vivo* assay using DTH reaction, Lewis rats	0.0029	[33]

*Selectivity for Kv1.3 channels (toxins unknown action on other channels)

^#^ Presented *in vivo* assays

EC_50_: half maximal effective concentration

IC_50_: half maximal inhibitory concentration

K_d_: dissociation constant

ND: not-determined

## Kv1.3 channels and their therapeutic implications

After presenting an in-depth view regarding Kv1.3 channel structure, function and mechanism on the immunological system, along with a list of all known scorpion toxins targeting this channel, this section will demonstrate channel effectiveness as a therapeutic approach. Although many scorpion toxins presented selective capacity to block Kv1.3 channels, most of them were only evaluated using *in vitro* tests (e.g. voltage clamp with two microelectrodes, patch-clamp, etc). Therefore, even though they can be classified as a potential candidate to therapeutic use, no evidence was reported *in vivo* and/or in humans. Thence, these toxins will not constitute the focus of this discussion. Below are presented few examples and a discussion supporting scorpion toxins that block Kv1.3 channel and present proof of concept *in vivo*.

An association was established between the expression of potassium channels, the proliferation and survival of oncotic cells. Nevertheless, it is not clear if these channels can participate in the angiogenic stimulation, checkpoint approval during mitosis or other mechanisms involved. Even though, it was observed that Kv1.3 channels overexpression leads to tumoral cell growth. Based on that, a trial facing A549 cell line (human lung adenocarcinoma) *in vitro* using margatoxin (toxin from *Centruroides margaritatus* scorpion venom) was done, resulting in suppression of lung carcinoma, achieving a significant blockage of the tumor grown and even a reduction on its volume. Another approach applied margatoxin into a xenograft model using nude mice and the toxin caused a reduction of tumor volume when it was injected into the tumor tissues [135, 136, 153].

A very promising toxin is OsK1 from *Orthochirus scrobiculosus* scorpion venom*,* more specially its synthesizable mutated form, which allows to improve its characteristic of being more specific to Kv1.3 than to Kv1.1 and 1.2 channels. The toxin has been tested *in vitro* upon L929 and murine erythroleukaemia (MEL) cells stably expressing mouse Kv1.3 (mKv1.3), Kv3.1 (mKv3.1), human Kv1.5 (hKv1.5) channels and COS7 cells, as well as using *in vivo* assays in C57/BL6 mice. These assays demonstrated a high inhibitory effect of the peptide and its analogues on Kv1.3 channels. Therefore, due to the inborn potential and the possibility of versatile analogues, OsK1 is considered an important peptide for the development of immunosuppressive drugs [143].

Knowing that Kv1.3 channel holds an essential role to regulate ions and function of immunological cells, theoretically, any peptide capable of suppressing the Kv1.3 channel is a potential therapeutic option for diseases that require immunosuppression. Thus, different trials have been conducted to define if the suppression of this channel could lead to selective immunosuppression resulting in benefits to a patient with autoimmune inflammatory diseases [18, 33, 149, 154, 155].

Tests with ImKTx88 (from *Isometrus masculatus* scorpion venom) were conducted with mice model of EAE to evaluate the toxin efficacy to prevent the blood-brain barrier disruption and subsequent infiltration of auto-reactive lymphocytes. This peptide was able to improve the severity of the disease and stabilize the barrier by selectively blocking Kv1.3 channels, leading to changes in expressions of adhesion molecules, receptors and interleukins. This effect is known as a recommendation of a possible therapy to multiple sclerosis [127, 156].

HsTX1 and its analogs, PEG-HsTX1[R14A] and HsTX1[R14A] were able to reduce inflammation in an active DTH model (Lewis rats) and in the pristine-induced arthritis rat model (Dark Agouti rats) [124].

Likewise, peptide Vm24, from *Vaejovis mexicanus smithi* scorpion venom, in murine models (female Lewis rats), was able to reduce the DTH reaction. Further studies showed the high affinity of the toxin with human lymphocytes, suggesting new experiments to determine possible clinical application [33, 157].

Kaliotoxin (from *Androctonus mauretanicus mauretanicus* scorpion venom) was able to decrease T-cell activation, leading to a decreased bone resorption when facing experimental periodontal disease in rat models. Thus, this toxin works as a factor of bone protection, being considered a potential therapeutic drug to prevent alveolar bone loss in humans [129, 158, 159].

Charybdotoxin (from *Leiurus quinquestriatus hebraeus* scorpion venom) suppressed lymphocyte proliferation and interleukin-2 (IL-2) production in both human and mice lymphocytes. However, because it is considered as a less-selective inhibitor, it can cause adverse effects, such as decrease in prothrombinase activity as well as exposure of phosphatidylserine (an outer surface membrane aggregating factor), leading to predisposition to hemorrhage due to the consumption of coagulation factors [16, 158, 160, 161].

Ts6 and Ts15 toxins (from *Tityus serrulatus* scorpion venom) demonstrated suppressive effects on DTH in BALB/c mice paw tissue 24h post-toxin challenge. DTH is a reaction mediated predominantly by skin-homing T_EM_ CD4^+^. Therefore, the study indicates that these toxins could be promising candidates for autoimmune disease therapy [148].

## Concluding remarks

The Kv1.3 channels had already proved their potential as a therapeutic target to treat diseases, such as cancer and autoimmune diseases. Since Kv1.3 regulates calcium signaling inside T_EM_ cells, which were considered the major actors in mediating chronic autoimmune response, molecules that present selectivity and high affinity to this channel could be used to design novel immunosuppressive drugs. A new medicine discovered from single venom could be seen as a gift by nature. Scorpion toxins are known to be a great source of neurotoxins including Kv1.3 blockers. With 68 promising Kv1.3 toxins’ blockers so far, it is evident that new immunosuppressive therapeutic drugs can be obtained by scorpion venoms. However, these molecules still need to be structurally improved (e.g. chemical modifications to improve selectivity and reduce immunogenicity) before reach the market, besides further *in vivo* and clinical studies.

### Abbreviations

ATP: adenosine triphosphate; Ca^2+^: calcium; CD25: interleukin-2 receptor alpha chain; CNS: central nervous system; CRAC: calcium-release-activated-calcium channel; CTLA4: cytotoxic T-lymphocyte-associated protein 4; DTH: delayed-type hypersensitivity; EAE: experimental autoimmune encephalomyelitis; FoxO1: forkhead box protein O1; GATA1: erythroid transcription factor; GYGD: glycine-tyrosine-glycine-aspartic acid; IL-2: interleukin-2; IL-10: interleukin-10; InsP3R: inositol-1,4,5-trisphosphate receptor; KCa: potassium channel activated by calcium; KTx: potassium channel acting toxins; Kv: voltage-gated potassium channels; Kv1: voltage-gated potassium channel type 1; Kv1.3: voltage-gated potassium channel type 1.3; K^+^: potassium; MCU: mitochondrial Ca^2+^ uniporter; NK: natural killer; PSD-95: postsynaptic density protein 95; PMCA: plasma membrane Ca^2+^ ATPase; pSTAT5: phosphospecific signal transducer and activator of transcription 5; SERCA: sarco/endoplasmatic reticulum Ca^2+^ ATPase; T_CM_: central memory T cells; T_EM_: T-effector memory lymphocytes; TrKB: Tropomyosin receptor kinase B; T_RM_: tissue resident memory T cells; T_SCM_: stem memory T cells. 
